# Comparison of Three Sample Preparation Procedures for the Quantification of L-Arginine, Asymmetric Dimethylarginine, and Symmetric Dimethylarginine in Human Plasma Using HPLC-FLD

**DOI:** 10.1155/2018/6148515

**Published:** 2018-02-01

**Authors:** Anne Marie Voigt Schou-Pedersen, Jens Lykkesfeldt

**Affiliations:** Experimental Pharmacology and Toxicology Laboratory, Department of Veterinary and Animal Sciences, Faculty of Health and Medical Sciences, University of Copenhagen, Ridebanevej 9, 1870 Frederiksberg C, Denmark

## Abstract

Increased asymmetric dimethylarginine (ADMA) in human plasma has been associated with reduced generation of nitric oxide, leading to atherosclerotic diseases. ADMA may therefore be an important biomarker for cardiovascular disease. In the present study, three sample preparation techniques were compared regarding the quantification of L-arginine and ADMA in human plasma: (A) protein precipitation (PP) based on aqueous trichloroacetic acid (TCA), (B) PP using a mixture of ammonia and acetonitrile, and (C) solid-phase extraction (SPE). The samples were analysed by using high-performance liquid chromatography with fluorescence detection (HPLC-FLD). The analytical performance of (A) was comparable with that of (C), demonstrating recoveries of >90%, coefficient of variations (CVs, %) of <8, and a resolution (*R*_*s*_) between ADMA and symmetric dimethylarginine (SDMA) of 1.2. (B) was disregarded due to recoveries below 75%. (A) was validated with good results regarding linearity (>0.994), precision (<5%), and sensitivity (lower limit of quantification (LLOQ)) of 0.14 *µ*M and 12 nM for L-arginine and ADMA, respectively. Due to the simplicity and speed of procedure (A), this approach may serve as preferred sample preparation of human plasma samples before HPLC-FLD in providing important information regarding elevated ADMA concentrations.

## 1. Introduction

Nitric oxide (NO) is important in numerous biological processes including the relaxation of smooth muscles and inhibition of platelet aggregation [1]. The endothelial nitric oxide synthase (eNOS) enzyme is involved in mediating vasodilation in vascular endothelium due to its role in the conversion of L-arginine to L-citrulline and NO, the latter inducing vasodilation. N^G^,N^G^-dimethyl-L-arginine (asymmetric dimethylarginine, ADMA) is a metabolic by-product from protein modification, and due to its similarity with L-arginine, ADMA is a competitive inhibitor of eNOS [2]. Increased concentrations of ADMA have been found to correlate with impaired endothelial vasodilation in several clinical studies possibly leading to the development of atherosclerotic diseases [3–5], and ADMA is considered to be an important biomarker. The similar N^G^,N^G′^-dimethyl-L-arginine, SDMA, is not an inhibitor of eNOS but competes with L-arginine in cellular uptake [6]. The reliability of ADMA as a biomarker of endothelial dysfunction is dependent on its precise analytical quantification, which is challenging due to the narrow concentration range found in healthy individuals [7].

A sample preparation step is often required before chromatographic analysis in order to separate the analytes of interest from proteins and other possible interferences in plasma [8]. Commonly used sample preparation techniques include PP, solid-phase extraction (SPE), and liquid-liquid extraction (LLE) [9]. Regarding the analysis of L-arginine, ADMA, and SDMA in human plasma, sample preparation by SPE using cation-exchange columns is the currently preferred approach [10–13]. A few publications exist for quantification of L-arginine, ADMA, and SDMA, where simple PP has been performed with 5-aminosalicylic acid [14], ethanol [15], or acetonitrile/ammonia [16]. However, these methods still consist of several steps including lyophilisation or evaporation.

After sample preparation, the separation and subsequent detection of L-arginine, ADMA, and SDMA are often performed using HPLC-FLD after pre- or postcolumn derivatization of L-arginine and ADMA with a fluorescent tag. In most studies, the fluorophore *ortho*-phthalaldehyde-mercaptoethanol (OPA-ME) has been used [13–15, 17, 18], but 6-aminoquinolyl-*N*-hydroxysuccinimidyl carbamate (AccQ Fluor reagent, Waters) has also been employed [11, 19]. Besides HPLC-FLD, additional separation and detection principles may come into play, for example, capillary electrophoresis coupled to UV [20, 21] or HPLC coupled to a mass spectrometer [22, 23]. Quantification based on commercial ELISA kits has been described lately, but may overestimate the concentration of ADMA, and is typically less precise [1].

Since current analytical methods described in the literature for quantification of L-arginine and ADMA involve relatively complicated and time-consuming sample preparation procedures, the aim of the present study was to investigate whether simple and fast PP before quantification of the biomarkers L-arginine, ADMA, and SDMA in human plasma may result in acceptable analytical performance evaluated by the FDA guideline for bioanalytical validation [24]. A partial validation of SDMA was performed, since L-arginine and ADMA were our primary goals of interest. Two different PP approaches were compared with state-of-the-art SPE cleanup regarding resolution, recovery, precision, simplicity, and cost. L-arginine, ADMA, and SDMA were labelled with the fluorescent tag, OPA-ME, and the derivatives were separated by using a reversed-phase HPLC-FLD method.

## 2. Materials and Methods

### 2.1. Instrumentation

All separations were performed on a Dionex Ultimate 3000 (Thermoscientific, Waltham, MA, USA) coupled to a Dionex fluorescence detector containing a micro flow cell (2 *µ*l). Chromeleon 7.2 was used for data acquisition and analysis.

### 2.2. Materials

L-arginine (>99%), N^G^,N^G′^-dimethyl-L-arginine di(*p*-hydroxyazobenzene-*p*′-sulfonate) (SDMA), perchloric acid (PCA), potassium tetraborate, *ortho*-phthalaldehyde (OPA), and 2-mercaptoethanol (ME) were obtained from Sigma-Aldrich (Steinheim, Germany). N^G^,N^G^-dimethyl-L-arginine hydrochloride (ADMA, >98%) was purchased from Cayman Chemical Company (Ann Arbor, MI, USA). Di-potassium hydrogen phosphate, potassium-dihydrogen phosphate, trichloroacetic acid (TCA), perchloric acid (PCA), hydrochloric acid (37%), ammonia (25%), *ortho*-phosphoric acid (85%), methanol, sodium hydroxide, and acetonitrile were purchased from Merck (Darmstadt, Germany). Oasis MCX mixed-mode SPE columns (30 mg, 1 ml) were kindly supplied by Waters Denmark.

### 2.3. Calibration Standards

Calibration stock solutions of 2.0 mM of L-arginine and 6.05 mM of ADMA and SDMA were prepared in 60% methanol and stored at −20°C until use. Working calibration standards of L-arginine, ADMA, and SDMA were prepared in 10 mM HCl in concentrations ranging from 0.5 *µ*M to 15 *µ*M for L-arginine and from 12 nM to 0.5 *µ*M for ADMA and SDMA. Calibration standards were analysed in duplicate.

### 2.4. Sample Preparation

Three different sample preparation techniques (A), (B), and (C) were evaluated by using human plasma obtained from one person. The procedures are briefly described in the following: (A) PP was performed on 50 *µ*l human plasma by adding 150 *µ*l cold 1 M TCA dissolved in water. After 10 min on ice, centrifugation was performed at 4°C with 16,000*g* for 10 min. The resulting supernatant was neutralised with equimolar amounts of 1 M aqueous NaOH and subsequently analysed. (B) PP was performed by adding 150 *µ*l of a cold mixture of ammonia and 25% acetonitrile (10  :  90, *v*/*v*) to 50 *µ*l human plasma. After 10 min on ice and centrifugation at 4°C with 16,000*g* in 10 min, the supernatant was analysed directly. (C) Oasis MCX SPE cartridges were initially washed with 300 *µ*l methanol and then with 300 *µ*l 0.1 M HCl. Fifty microliters of human plasma was diluted with 150 *µ*l of 4% (*v*/*v*) phosphoric acid and loaded onto the SPE. The SPE cartridge was then washed with 300 *µ*l of methanol and 300 *µ*l of 0.1 M HCl. The analytes were eluted with 400 *µ*l of a mixture of ammonia and 25%/1 M NaOH/water/MeOH (10  :  0.5  :  40  :  50, *v*/*v*). The solvent was removed by evaporation with nitrogen at a temperature of 60°C to 70°C. The residue was dissolved in 50 *µ*l of 60% methanol and analysed.

Recoveries in % were determined for each sample preparation technique by fortifying the concentration of L-arginine and ADMA in a human plasma sample with calibration standard and measuring the concentration in the spiked sample in relation to the concentration measured in the unspiked sample plus the added calibration standard concentration. The retention times for L-arginine, ADMA, and SDMA were verified in each sample preparation procedure by 10 times fortifications with calibration standards.

### 2.5. Chromatographic Separation and Detection

In order to detect L-arginine, ADMA, and SDMA, precolumn derivatization was performed automatically by mixing 3 *µ*l of borate buffer (400 mM, pH 10) with 1 *µ*l of 75 mM OPA dissolved in methanol and 1 *µ*l of 56 mM aqueous ME at 4°C. The mixture of sample and reagents was incubated at 4°C for 10 s. Of the resulting mixture, 3 *µ*l was injected onto the column.

The chromatographic conditions used in the current method were inspired by a previously published method for the quantification of L-arginine, ADMA, and SDMA [13]. Separation of the OPA-ME derivatives of L-arginine, ADMA, and SDMA was performed at a temperature of 40°C using a Phenomenex Gemini C_18_ column with the dimension 150 × 4.6 mm and a particle size of 5 *µ*m. Prior to the analytical column, a C_18_ guard column with the dimension 4 × 3 mm was placed. The mobile phase A consisted of 50 mM potassium phosphate (pH 6.5) containing 14% (*v*/*v*) acetonitrile. The mobile phase B was 60% (*v*/*v*) acetonitrile. Isocratic separation was performed with 100% mobile phase A at a flow rate of 1.2 ml/min. After 20 min, the mobile phase was switched to 100% B in 3 min to wash off late-eluting compounds. Finally, the column was re-equilibrated for 7 min giving a total run time of 30 min. The fluorescent OPA-ME derivatives of L-arginine, ADMA, and SDMA were detected at an excitation wavelength of 340 nm and an emission wavelength of 455 nm. Retention times for the L-arginine, ADMA, and SDMA derivatives were 10.5, 18.1, and 19 min, respectively.

### 2.6. Method Validation

Robustness was initially tested for procedure (A) applying different time intervals (2, 8, 10, and 12 min) for performing PP. Thereafter, the selected sample preparation technique, procedure (A), combined with the developed chromatographic method was validated with respect to linearity, recovery, precision, and sensitivity (taken as LLOQ) on a human plasma sample [24]. The recovery was tested at three concentration levels corresponding to approximately 30, 60, and 90% of the initial concentration of L-arginine and ADMA. The average recovery should be within 15% of the nominal value. Intra-assay variability was tested on human plasma (*n* = 6). Interassay variability was evaluated by analysing human plasma samples in triplicate on three different days (*n* = 9). A coefficient of variation (CV, %) of up to 15% was considered acceptable. LLOQ was estimated for L-arginine and ADMA by diluting human plasma with PBS buffer (pH 7.4) to the expected LLOQ, and recovery and precision was determined on these samples (*n* = 6). A precision and recovery range of no more than 20% variation was considered to be acceptable at LLOQ [24]. Upper limit of quantification (ULOQ) was taken as the highest concentration from the calibration curve.

The stability for extracted human plasma samples and calibration standards was tested for L-arginine and ADMA at 4°C. The long-term stability was further assessed for human plasma at −80°C and for calibration stock solutions at −20°C. All stability tests were performed in triplicate. Acceptable stability means that at least 90% of the initial amount was found at a given time point.

### 2.7. Quality Control Samples

Blood samples were obtained from six apparently healthy individuals after informed consent but without collection of any data on the individuals. The use of human blood for quality control purposes is not subject to ethical approval in Denmark. The blood was collected in Vacutainer^TM^ collection tubes containing K_3_-EDTA from Becton Dickinson (Franklin Lakes, NJ, USA) and centrifuged at 2000 g for 5 min at 4°C. The resulting plasma was stored in aliquots at −80°C and used for verification of the analytical method.

## 3. Results

### 3.1. Optimisation of Sample Preparation Procedures

Preliminary studies of different PPs were performed. Zinellu et al. showed that the recoveries of L-arginine and ADMA were low using 100% acetonitrile [21]. Acetonitrile containing TCA, PCA, or ammonia was tested. Furthermore, aqueous solutions of TCA and PCA were investigated. PP with PCA was not found to extract L-arginine and ADMA very efficiently as opposed to aqueous TCA, which showed promising results. However, decreasing concentrations of L-arginine and ADMA were found in the sample solutions, and a neutralization step was therefore added. Different ratios of 25% ammonia and acetonitrile (10  :  90, 20  :  80, and 30  :  70, *v*/*v*) were tested, but increasing the percentage of ammonia did not improve recovery markedly; thus, the 10  :  90 (*v*/*v*) ratio was retained, procedure (B).

In the development of the SPE cleanup, (C), adding PBS buffer (pH 7.4) to plasma in a ratio of 3  :  1 as described in previous studies was found to clog the Oasis MCX column, and therefore, human plasma was diluted with 4% phosphoric acid as described by the manufacturer's protocol. According to a recent publication by de Jong and Teerlink [25], the elution solvent for L-arginine and ADMA using Oasis MCX SPE cartridges was optimised to a mixture of concentrated ammonia/1 M NaOH/water/methanol (10  :  0.5  :  40  :  50, *v*/*v*), and this elution mixture was selected for the current SPE cleanup.

### 3.2. Results from Sample Preparation Procedures (A), (B), and (C)

#### 3.2.1. Recovery and Precision

Working calibration standards of L-arginine and ADMA dissolved in 10 mM HCl were used to construct a calibration curve for the comparison of the three sample preparation techniques (A), (B), and (C). Recoveries in % for L-arginine and ADMA were determined and are shown in Table 1. The average recoveries obtained for procedures (A) and (C) were higher than 90% for both L-arginine and ADMA, and therefore acceptable. Furthermore, procedure (A) showed satisfactory recoveries at three concentration levels. Procedure (B) demonstrated recoveries of less than 75%. The precision expressed as “CV, %” was estimated from the recovery experiments and was found to be less than 8% for the three tested sample preparation procedures (A–C).

#### 3.2.2. Chromatographic Performance

**Figure 1:**
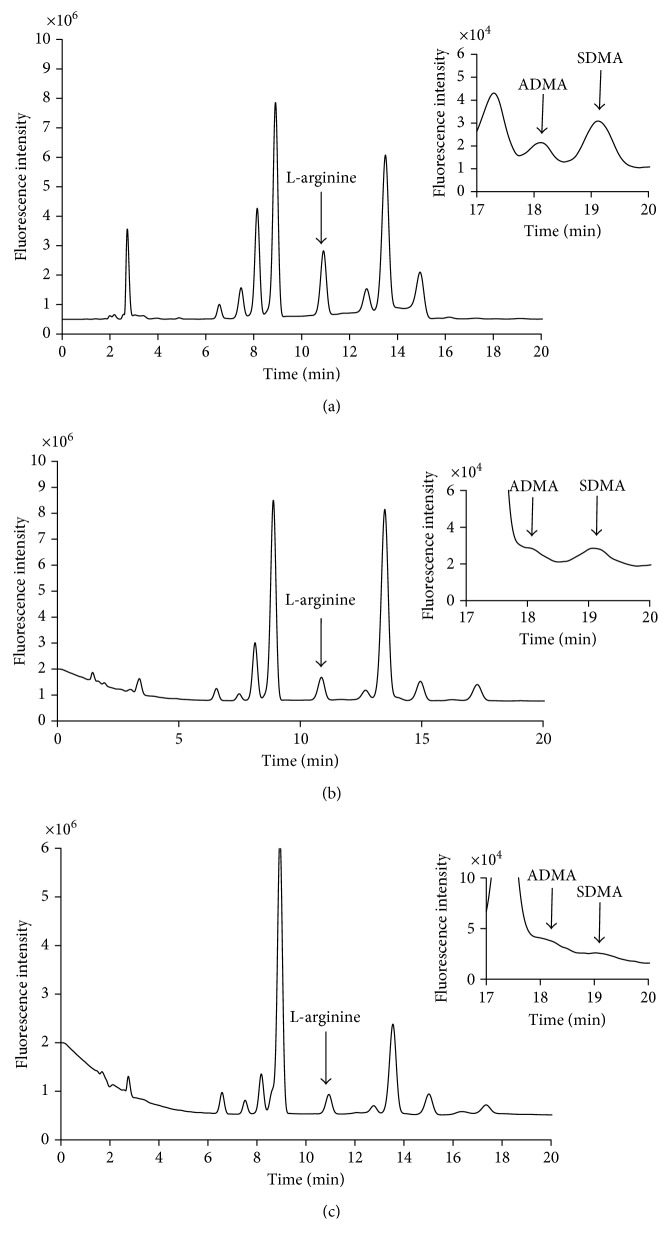
Representative chromatograms obtained from the analysis of human plasma using (a) procedure (A), PP with aqueous TCA; (b) procedure (B), PP with a mixture of acetonitrile and ammonia (90  :  10); and (c) procedure (C), SPE cleanup with Oasis MCX. Insets: zoom of the ADMA and SDMA peaks at 18.1 and 19.0 min, respectively.

Figures 1(a) show examples of chromatograms obtained after each of the three different sample preparation procedures (A), (B), and (C). No major differences were observed between the three obtained chromatograms. The resolution between ADMA and SDMA in procedure (A) was determined to be 1.2 as compared to the two other procedures, where *R*_*s*_ was below 1.

#### 3.2.3. Validation of Procedure (A)

The robustness of procedure (A) was initially tested by performing PP on ice with different time points (2, 8, 10, or 12 min). The robustness study did not show any difference between the obtained concentrations of L-arginine and ADMA; thus, this step was not found to be critical.

Validation results can be found in Tables 1 and 2. Obtained recoveries were from 91.5 ± 3.03% up to 113 ± 8.21%. Intra-assay variation (CV, %) was 3.2 for L-arginine, 3.2% for ADMA, and 4.2% for SDMA, whereas interassay variation was observed to be 5.0% for L-arginine, 3.3% for ADMA, and 3.8% for SDMA. At LLOQ, the found recoveries were 114% for L-arginine and 92% for ADMA and the intra-assay variations were 21% for L-arginine and 19% for ADMA (*n* = 6) (data not shown). L-arginine, ADMA, and SDMA were quantified in quality control samples using procedure (A) and the developed HPLC method. An average concentration of 64.1 ± 10.3 *µ*M for L-arginine, 0.27 ± 0.02 *µ*M for ADMA, and 0.57 ± 0.09 *µ*M for SDMA was obtained (*n* = 6).

Table 3 shows the stability at 4°C of a calibration standard prepared at ULOQ and of human plasma after extraction with procedure (A). Storage of calibration standards at 4°C is not recommended for more than 24 hours, whereas the stability of prepared human plasma at 4°C is acceptable for up to 48 h. Human plasma stored at −80°C and calibration stock solutions stored at −20°C are considered to be stable for at least 6 months.

## 4. Discussion

### 4.1. Comparison of Sample Preparation Procedures (A), (B), and (C)

In all three sample preparation procedures, baseline resolution could not be obtained between ADMA and SDMA, and both ADMA and SDMA were surrounded by larger unknown peaks. Optimisation of the HPLC method could possibly have improved the resolution, especially in the chromatogram obtained after performing procedure (C). However, in order to compare the sample preparation procedures, it was considered necessary to use exactly the same chromatographic method for all three procedures. Since baseline resolution was not attained, there is a chance of experiencing interference from adjacent peaks. However, the average concentration of ADMA found in the quality control samples did not indicate interference, since the observed concentrations were in the lower end of the ADMA range stated in literature [26]. Furthermore, the precision of the current method was very good (CV < 5%). In addition, it was possible to separate ADMA from SDMA with acceptable resolution, supporting that a reliable analytical method was developed.

The selectivity was surprisingly not markedly increased by performing the SPE cleanup (Figure 1(c)), although the observed unknown peaks are lower as compared to Figure 1(a). The minor improvement in selectivity obtained by the SPE cleanup may be explained by the limited capacity of SPE cartridges.

The supernatants obtained after performing procedures (A) and (C) were clear, as opposed to the relatively cloudy solution obtained after procedure (B), which further lead to recoveries below 75%. Zinellu et al. used a 10  :  90 mixture of ammonia  :  acetonitrile for PP. However, they performed additional steps of filtration and concentration of their samples, which allegedly improved the recovery.

The main difference between sample preparation procedures (A) and (B) in relation to (C) is that laborious steps in procedure (C) have to be performed, resulting in a diminished throughput of samples per day. This is not desirable in a clinical laboratory, where research studies often comprise many samples. In addition, the probability of introducing errors and the risk of analyte loss during performance of the several steps in the SPE cleanup makes this approach less preferable. Furthermore, the cost-effectiveness of (C) with acquisition of SPE cartridges and an increased workload in the laboratory are estimated to be around 10 Euros per sample, which is less attractive when compared to (A) with an estimated cost of 3 Euros per sample.

One argument often stated for performing the laborious SPE cleanups is the decreased contamination of the HPLC column and/or detector. However, when using procedure (A) and the developed analytical method including a washing step of the column, the pressure increase was not considered to be critical (around 5 bars per 100 human plasma samples) and could be eliminated by changing the precolumn. The same HPLC column has been used for several hundred human plasma samples without deterioration of the resolution.

### 4.2. Validation of Sample Preparation Procedure (A)

Overall, the validation results were acceptable. The recoveries for L-arginine were generally found to be above 100%. The reason for this could be that the calibration standard used for spiking in the recovery experiment was dissolved in 10 mM HCl, which apparently affected the observed recovery of L-arginine. However, the level of L-arginine in human plasma has not been observed to deviate from literature values; thus, the issue is considered to be related to the setup of the recovery experiment. Regarding LLOQ, the found recovery for L-arginine was just above the acceptance criterion of 20%. However, since the LLOQ for L-arginine is well below the expected concentration of L-arginine in human plasma, the marginally higher variation for L-arginine at LLOQ is not anticipated to have any practical importance.

L-arginine, ADMA, and SDMA concentrations for the quality controls can be found in Table 2 (*n* = 6). In the literature, the L-arginine concentration in human plasma has been reported with an interval of 59.4 to 95.6 *µ*M (*n* = 1141) [27]. Horowitz and Heresztyn have reported ADMA mean concentrations from 0.30 to 2.38 *µ*M in plasma or serum using different analytical techniques [26]. Teerlink reported ADMA and SDMA in plasma from 2311 patients with an average concentration of 0.50 *µ*M for ADMA and 0.53 *µ*M for SDMA. 95% confidence intervals for ADMA and SDMA were reported to 0.39–0.63 *µ*M and 0.38–0.73 *µ*M, respectively [7]. The obtained results for the quality control samples for L-arginine are within the reported range, although in the lower end. The found ADMA and SDMA concentrations in the current study are close to the literature values, although a bit lower for ADMA. However, the sample size was also quite small.

## 5. Conclusions

The analytical performance of sample preparation procedure (A) consisting of simple PP with TCA is considered to be superior to procedure (B) and similarly as good as performing a SPE cleanup, procedure (C) in quantifying L-arginine, ADMA, and SDMA in human plasma using HPLC-FLD. The developed HPLC-FLD method with sample preparation procedure (A) proved to be both accurate with recoveries ranging from 91.5 ± 3.03% to 113 ± 8.21% and precise with CVs of no more than 5%. Furthermore, the sensitivity was acceptable with an LLOQ of 0.14 *µ*M for L-arginine and 12 nM for ADMA. The easy sample preparation in our analytical method allows for high throughput of samples from clinical research studies in the quantification of L-arginine, asymmetric dimethylarginine (ADMA), and symmetric dimethylarginine (SDMA) in plasma. ADMA is important in the preliminary evaluation of endothelial dysfunction in humans.

## Figures and Tables

**Table 1:** Determined average concentrations of L-arginine and ADMA in human plasma ±SD (*n* = 3) and average recovery ±SD (*n* = 3) at three concentration levels obtained with sample preparation procedure (A). Recovery values in % given as mean ± SD (*n* = 3) obtained for procedure (B) and procedure (C) are shown for comparison.

	Human plasma, mean ± SD (*µ*M)	Procedure A			Procedure B	Procedure C
		Added (*µ*M)	Measured, mean ± SD (*µ*M)	Recovered, mean ± SD (%)	Recovered, mean ± SD (%)	Recovered, mean ± SD (%)
L-arginine	65.3 ± 2.09	20	94.3 ± 10.1	113 ± 8.21	—	—
		40	115 ± 3.20	109 ± 2.78	68.4 ± 5.50	118 ± 3.62
		60	138 ± 8.67	110 ± 6.92	—	—
ADMA	0.40 ± 0.013	0.1	0.47 ± 0.030	91.5 ± 3.03	—	—
		0.2	0.59 ± 0.070	98.0 ± 7.07	73.5 ± 4.22	94.7 ± 8.46
		0.3	0.70 ± 0.032	98.9 ± 4.55	—	—

**Table 2:** Validation data: calibration curve equation, correlation coefficient, LLOQ, ULOQ, intra- and interday precision, and average concentration ± SD quality control samples (human plasma from 6 volunteers) for L-arginine and ADMA using the selected sample preparation (A) with the developed chromatographic method.

	Calibration curve equation	Correlation coefficient (*R*^2^)	LLOQ (*µ*M)	ULOQ (*µ*M)	Intraday precision (CV, %) (*n* = 6)	Interday precision (CV, %) (*n* = 9)	Quality control (*µ*M) (*n* = 6)
L-arginine	*y* = 78686*x* − 3519	0.999	0.14	15	3.2	5.0	64.1 ± 10.3
ADMA	*y* = 73655*x* + 1134	0.994	0.012	0.50	3.4	3.3	0.27 ± 0.02
SDMA	*y* = 87003*x* + 3261	0.989	—	—	4.2	3.8	0.57 ± 0.09

**Table 3:** Stability data for human plasma prepared by procedure (A) and a calibration standard at ULOQ as well as long-term data at −80°C for human plasma and at −20°C for a calibration stock solution. The data are calculated as average of three determinations.

	4°C				−80°C	−20°C
	Prepared human plasma		Calibration standard		Human plasma	Calibration stock solution
	24 h	48 h	24 h	48 h	6 months	
L-arginine	96.1%	95.4%	91.1%	87.5%	95.3%	91.5%
ADMA	92.8%	93,5%	91.8%	89.6%	95.0%	103%
